# Unexpected cholinergic crisis caused by distigmine bromide: A case report

**DOI:** 10.1097/MD.0000000000031677

**Published:** 2022-11-25

**Authors:** Toshiki Sera, Shinji Kusunoki, Nobuaki Shime

**Affiliations:** a Department of Emergency and Critical Care Medicine, Graduate School of Biomedical and Health Sciences, Hiroshima University, Minami-ku, Hiroshima, Japan; b Critical Care Medical Center, Hiroshima Prefectural Hospital, Minami-ku, Hiroshima, Japan.

**Keywords:** cholinergic crisis, distigmine bromide

## Abstract

Distigmine bromide is a cholinesterase (ChE) inhibitor used to treat dysuria due to a hypotonic bladder. We encountered a case of cholinergic crisis caused by distigmine bromide, which resulted in a rapid decrease in serum ChE levels, hypothermia, respiratory failure, and circulatory failure within a short period of time.

**Patient concerns:**

A 51-year-old man was admitted to a psychiatric hospital to treat behavioral disorders due to irritability and violent behavior. The patient was referred to our hospital for septic shock secondary to urinary tract infection and respiratory failure. He had not defecated for 5 days before visiting our hospital. He had moderate intellectual disability. Immediately after admission, he developed hand tremors and drooling. The airway was obstructed by drooling due to vomiting of yellow clear gastric juice.

**Diagnosis:**

The patient’s high saliva volume, bradycardia, respiratory failure (54 breaths/min), constricted pupils (2.5/mm), poor oxygenation, and a history of oral medication were consistent with the diagnosis of cholinergic crisis due to distigmine bromide.

**Interventions:**

On admission, the patient was immediately intubated. He was treated with noradrenaline (0.1 µg/kg/min) to increase his blood pressure. He was admitted to the intensive care unit (ICU). Since he had circulatory failure, vasopressin (approximately 1 U/h) was administered. Continuous intravenous atropine sulfate (0.6 mg/h) was also administered for high saliva volume.

**Outcomes:**

On the 8th ICU day, the patient’s drooling and bradycardia improved. The patient was physically and mentally stable, and transferred to the referring hospital.

**Lessons:**

ChE levels and symptoms before onset may not be useful for the early detection and prevention of adverse effects of cholinergic crisis caused by distigmine bromide. In addition to known risks such as renal impairment and older age, constipation should be recognized and communicated as a risk factor.

## 1. Introduction

Cholinergic crisis is caused by overstimulation of nicotinic and muscarinic receptors at the neuromuscular junction. In clinical practice, a common cause is overmedication with acetylcholine esterase inhibitors which are used for the treatment of myasthenia gravis, reversal of neuromuscular blockage, and exposure to organophosphates. Both muscarinic and nicotinic toxicity symptoms are induced. These symptoms include convulsions, increased salivation, weeping, muscle weakness, paralysis, muscle contractions, and diarrhea. Although data on the epidemiology of this disease are scarce, the mortality rate is 3% to 25%, and the most common cause of death is progressive respiratory failure.^[1]^

Distigmine bromide is an acetylcholine esterase inhibitor which is used for dysuria caused by a hypotonic bladder,^[2]^ with cholinergic crisis as a serious side effect. In 2010, the package insert approved by the Japanese Ministry of Health, Labour, and Welfare was revised, and the dosage was set at 5 mg/day. We encountered a case of sudden decrease in serum ChE (cholinesterase) and development of severe cholinergic crisis in an elderly patient, who was not considered at risk of developing a cholinergic crisis. No similar cases have been previously reported.

## 2. Case report

A 51-year-old man with a body mass index of 23.4 was referred to our emergency center. His medical history included moderate intellectual impairment and neurogenic bladder (self-directed urination). He had been taking distigmine bromide (5 mg/day for approximately 4.5 years), sodium valproate, carbamazepine, urapidil, bethanechol chloride, risperidone, lemborexant, quazepam, and levomepromazine maleate.

He was admitted to a psychiatric hospital to treat behavioral disorders due to difficulties at the support facility for independent living (irritability and violent behavior). The patient was referred to our hospital for septic shock secondary to urinary tract infection and respiratory failure. He had not defecated for 5 days before visiting our hospital. He had moderate intellectual disability and no relevant family history of the disease. Blood tests performed on the morning of the patient’s transfer (9 h before referral) showed normal serum ChE (198 IU/L) and renal function (Table 1). There were no abnormalities until 6 h before referral to our hospital. He subsequently developed hand tremor, drooling, poor oxygenation, hypothermia of 34°C, and hypotension. At that time, the blood test results showed decreased serum ChE levels (Table 1). Serum ChE was performed as part of routine testing in a patient with such a deteriorating condition.

**Table 1:** Blood chemistry before admission to our hospital.

	9 h before admission	2h before admission	
Total bilirubin	0.3	0.3	mg/dL
Aspartate aminotransferase	17	22	IU/L
Alanine aminotransferase	11	15	IU/L
Lactate dehydrogenase	148	195	IU/L
Cholinesterase	198	23	IU/L
Creatine phosphokinase	15	Not done	IU/L
Total protein	6.7	8.0	g/dL
Albumin	3.2	3.9	g/dL
Blood urea nitrogen	6.3	9.9	mg/dL
Creatinine	0.76	1.16	mg/dL
C-reactive protein	0.3	Not done	mg/dL

**Figure 1.**
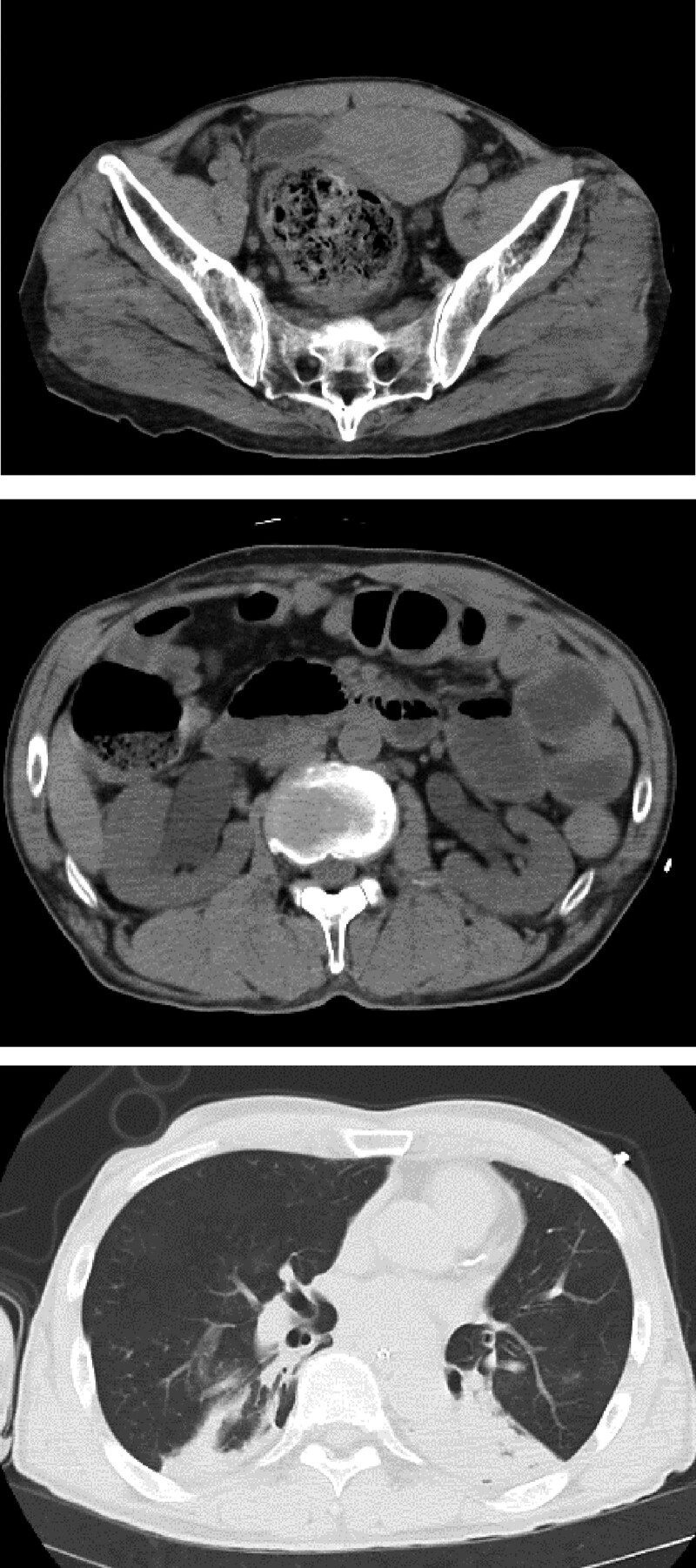
Computerized tomography findings at the time of admission. Computerized tomography showed bilateral hydronephrosis (no urinary tract obstruction), severe constipation, and bilateral atelectasis, probably due to salivary dribbling.

Immediately after admission, he developed hand tremors and drooling. The airway was obstructed by drooling due to vomiting of yellow clear gastric juice. Vital signs were as follows: respiratory rate = 30 breaths/min; SpO_2_ 72% (under hyperoxia and bag mask ventilation), blood pressure = 73/38 mm Hg, heart rate = 67 beats/min, Glasgow coma scale = E2V2M5, pupils, 3.5 mm bilaterally with light reflex, and body temperature 32.5°C. The patient was immediately intubated, noradrenaline (0.1 µg/kg/min) was administered to increase his blood pressure, and he was admitted to the intensive care unit (ICU). Arterial blood gas analysis showed pH = 7.23, PaCO_2_ = 49.4 mm Hg, PaO_2_ = 53.6 mm Hg (F_I_O_2_ = 1.0), HCO_3_-= 24.9 mEq/L, and lactate = 3.2 mmol/L under intubation (FiO2 1.0). Computerized tomography revealed bilateral hydronephrosis with no urinary tract obstruction, severe constipation, and bilateral lung atelectasis (Fig. 1).

**Figure 2.**
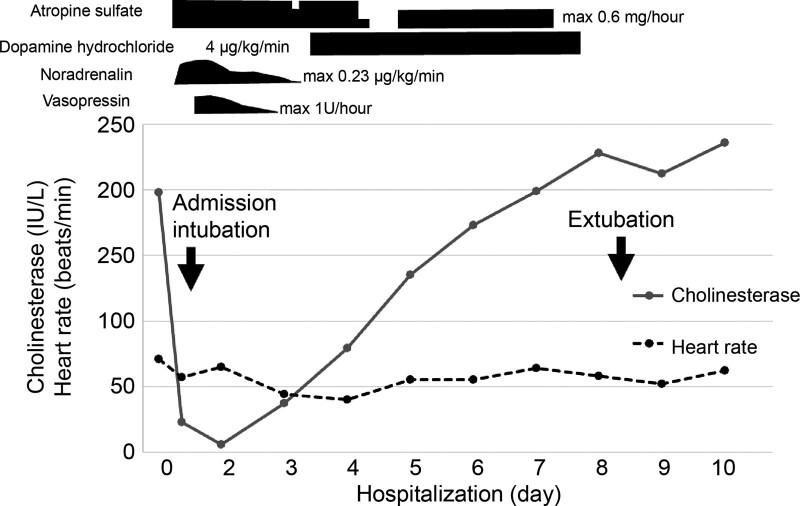
Clinical course.

Circulatory failure necessitated the use of vasopressin (approximately 1 U/h) in combination with noradrenaline (Fig. 2). High saliva volume, bradycardia, respiratory failure (54 breaths/min), constricted pupils (2.5/mm), poor oxygenation, and a history of oral medication were consistent with the diagnosis of cholinergic crisis due to distigmine bromide. After initiating continuous intravenous atropine sulfate (0.6 mg/h), saliva volume gradually decreased, and noradrenaline and vasopressin could be terminated on the third ICU day. On the fourth ICU day, atropine sulfate was tapered down due to suspected decreased gastrointestinal peristalsis, resulting in bradycardia of 30 beats/min and hypotension (systolic blood pressure: approximately 70–80 mm Hg, diastolic blood pressure: approximately 40–50). Therefore, tapering off atropine sulfate was completed while continuous intravenous dopamine (4 μg/kg/min) was administered. However, drooling gradually reappeared and was judged to be a possible obstacle to extubation; therefore, atropine sulfate (0.4 mg/h) was resumed on the 6th day. On the 8th day, the patient’s drooling and bradycardia improved, and both medications were discontinued. The patient was physically and mentally stable, and transferred to the referring hospital. Written informed consent was obtained from the patient for the publication of this Case report.

## 3. Discussion

We encountered a case of cholinergic crisis caused by distigmine bromide administration. Risk factors for the development of cholinergic crisis with distigmine bromide include early cases within the first 2 weeks of administration, daily doses > 10 mg, distigmine doses > 0.1 mg/kg/day, elderly patients, fasting, and impaired renal function.^[3–7]^ This patient had a history of long-term oral medication use, was not at risk of developing crisis, and had normal ChE levels and renal function before this episode.

The blood concentration of distigmine remained abnormal 24 h after the patient stopped taking it, and was presumed to have been involved in the development of the crisis (Table 2). Distigmine levels normalized by day 3, but severe symptoms persisted for approximately 1 week. Approximately 85% of distigmine is renally excreted,^[8]^ but there have been reports of intestinal obstruction and paralytic ileus triggering a crisis.^[9,10]^ Intestinal obstruction may cause an increase in the blood concentrations of orally administered drugs.^[9]^ Our patient did not defecate for 4 days, and no dehydration or renal dysfunction was detected, leading to a suspicion of constipation as a presumed cause of the elevated distigmine blood levels.

**Table 2:** Transition of serum cholinesterase and distigmine bromide concentrations.

Day	Time	Cholinesterase (IU/L)	Distigmine bromide (ng/mL)
−25		171	ー
1	8:00	198	ー
1	15:00	23	ー
2	6:00	6	40.4
3	6:00	37	.3
4	6:00	79	<.2
5	6:00	135	<.2
6	6:00	173	<.2
7	6:00	199	<.2

Low serum ChE level is considered a risk predictor for adverse drug reactions to distigmine, and the dose, older age, and progression of chronic kidney disease may confer additional risk.^[7]^ Kobayashi et al^[9]^ reported prodromal symptoms such as noticeable drooling, a parasympathetic symptom, 1 month before an event.^[10]^ However, as in the present case, CHE levels can drop rapidly and symptoms can appear rapidly within a few hours. Hence CHE levels can be used for definitive diagnosis but are not likely to be used for prevention of a crisis. In light of this, it seems to be most important for patients taking distigmine to avoid constipation, in addition to avoidance of other known risk factors.

## 4. Conclusion

We encountered a case of cholinergic crisis caused by distigmine bromide administration. ChE levels and symptoms before onset may not be useful for early detection and prevention of adverse effects. Constipation may be recognized as a risk factor of intoxication.

## Acknowledgments

I wish to thank TORII PHARMACEUTICAL CO., LTD, for measuring the blood levels of distigmine. And we would like to thank Editage (www.editage.com) for English language editing.

## Author contributions

**Conceptualization:** Toshiki Sera.

**Data curation:** Toshiki Sera.

**Methodology:** Toshiki Sera.

**Project administration:** Nobuaki Shime, Shinji Kusunoki.

**Supervision:** Nobuaki Shime, Shinji Kusunoki.

**Writing ‐ original draft:** Toshiki Sera.

**Writing ‐ review & editing:** Toshiki Sera, Nobuaki Shime, Shinji Kusunoki.
